# DLL4 overexpression increases gastric cancer stem/progenitor cell self‐renewal ability and correlates with poor clinical outcome via Notch‐1 signaling pathway activation

**DOI:** 10.1002/cam4.962

**Published:** 2016-11-28

**Authors:** Zhi‐Feng Miao, Hao Xu, Hui‐Mian Xu, Zhen‐Ning Wang, Ting‐Ting Zhao, Yong‐Xi Song, Ying‐Ying Xu

**Affiliations:** ^1^ Department of Surgical OncologyFirst Hospital of China Medical UniversityShenyangLiaoning ProvinceChina; ^2^ Department of Breast SurgeryFirst Hospital of China Medical UniversityShenyangLiaoning ProvinceChina

**Keywords:** Cancer stem/progenitor cells, DLL4 expression, drug resistance, prognosis, stomach cancer

## Abstract

Gastric cancer is one of the most common malignant diseases, and poses a serious threat to the quality of human life. Gastric cancer stem/progenitor cells (GCSPCs) have critical effects on tumor formation, affecting specific features of self‐renewal and differentiation and playing a critical role in metastasis. The Notch‐1 pathway is crucially important to GCSPCs and is regulated by DLL4. In this study, DLL4 and Nestin levels were measured in 383 gastric cancer tissue samples by immunohistochemistry, and the clinico‐pathological features of patients assessed. After DLL4 silencing in selected gastric cancer cell lines, the expression of GCSPC markers and colony formation ability were analyzed and the self‐renewal and differentiation capacities of the cells were evaluated. The relationship between DLL4 levels and Notch‐1 signaling pathway effector amounts was assessed via Western blotting and immunofluorescence. Finally, the tumor formation ability of the gastric cancer cells was evaluated with different levels of DLL4 and multiple cell densities in vivo. Our results indicate that DLL4 expression is associated with TNM stage and cancer metastasis, with high amounts of DLL4 leading to poor outcome. DLL4 silencing inhibited the self‐renewal ability of GCSPCs and increased their multidifferentiation capacity, resulting in reduced GCSPC ratios. DLL4 knockdown also blocked the Notch‐1 pathway, weakening invasion ability and resistance to 5‐FU chemotherapy. In vivo, DLL4 silencing inhibited the tumor formation ability of GCSPCs. In conclusion, DLL4 affects GCSPC stemness, altering their pathological behavior. DLL4 silencing inhibits GCSPC metastatic potential both in vitro and in vivo by impeding Notch‐1 signaling pathway activation, indicating that DLL4 may be a new potential therapeutic target.

## Introduction

Gastric cancer is one of the most common malignant diseases around the world, and presents a serious threat to human health 1, 2. With the continuous improvement of clinical diagnostic procedures, early‐stage gastric cancer is easier to detect than before, allowing for moderately satisfactory therapy. In contrast, patients with advanced gastric carcinoma – especially those in the metastatic stage – still present poor prognosis despite systematic operative treatment and chemotherapy 3, 4.

Multiple studies have investigated the mechanisms of gastric cancer metastasis 5, 6, 7. Recent reports focused on the effects that the tumor environment has on gastric cancer stem/progenitor cell (GCSPC) stemness, indicating that it is critical during the GCSPC metastasis process. As a burgeoning field, cancer stem/progenitor cells (CSPCs) research have recently attracted increasing. CSPCs have potent effects on tumor formation via specific features of self‐renewal and differentiation, play important roles in the progress of gastric cancer metastasis, and worsen prognosis 8, 9. Although the exploration of CSPCs expanded our knowledge of tumor incidence and development, their specific mechanisms need further investigation.

Delta‐like ligand 4 (DLL4) is one of the ligands that regulate the activities of Notch pathways, including Notch‐1, 2, and 4. DLL4 expression highly correlates with tumor angiogenesis and metastasis 10. Studies have indicated that the DLL4‐Notch system plays a critical role in tumor neovascularization and causes productive angiogenesis in some malignancies such as nasopharyngeal carcinoma and pancreatic cancer 11, 12. As recently reported, the Notch‐1 pathway also plays a crucial role in GCSPC pathology by mediating the proliferation and differentiation of CSPCs 13. However, whether DLL4 expression influences GCSPC metastatic potential remains largely unknown.

In this study, GCPSCs were obtained from several gastric cancer cells lines. The correlation between DLL4 expression and the clinico‐pathological features of gastric cancer patients was then assessed. Next, we verified that DLL4 expression affected GCSPC function by regulating activity of the Notch‐1 pathway, driving cancer metastasis and resulting in unfavorable disease outcome. This enriches our understanding of the mechanisms of gastric cancer metastasis and may provide a new therapeutic target in clinical oncology.

## Materials and Methods

### Tissue samples

Tumor specimens were obtained from 383 patients with gastric cancer who underwent radical surgery at the Department of Surgical Oncology, First Hospital of China Medical University, from 2006 to 2009. All patients underwent R0 gastrectomy, and their clinical and pathological data were available. Surgical specimens were examined by experienced pathologists, and the distal resection margins were tumor‐free. The distant metastasis data were collected during postoperation follow‐up. They have concrete evidence for metastasis/recurrence organ which were confirm by computed tomography. The study protocol was reviewed and approved by the Ethics Committee of China Medical University, and all patients provided written informed consent.

### Immunohistochemical staining of tissue samples

Sections were deparaffinized with xylene and rehydrated through a graded ethanol series. The sections were then boiled for 10 min in 0.01 mol/L citrate buffer, and endogenous peroxidase activity was blocked with 0.3% H_2_O_2_ in methanol for 30 min. After overnight incubation at 4°C with rabbit polyclonal anti‐DLL4 antibodies (diluted 1:1000; Abcam, Cambridge, MA), mouse monoclonal anti‐Nestin antibodies (diluted 1:500; Chemicon, Temecula, CA) or rabbit polyclonal anti‐NICD‐1 antibodies (diluted 1:400; CST, USA), the sections were exposed to biotin‐labeled secondary antibodies for 1 h and developed with DAB‐H2O2. Staining was scored using the following scale: 0, no staining; 1+, minimal staining; 2+, moderate to strong staining in at least 20% of cells; 3+, strong staining in at least 50% of cells. Cases with 0 or 1+ staining were classified as negative; those with 2+ or 3+ staining were considered to be positive. Human Tubulin staining was deemed as positive control and sample stain with secondary antibody alone was deemed as negative control.

### Cell culture

The gastric cancer cell (GCC) lines SGC 7901, BGC 823, HGC 27, and Kato III were obtained from the Department of Cell Biology, China Medical University, China. They were cultured in Dulbecco's Modified Eagle's medium (DMEM; Gibco, Carlsbad, CA) supplemented with 10% fetal bovine serum (FBS; Gibco), 100 units/mL penicillin, and 100 *μ*g/mL streptomycin. Human Umbilical Vein Endothelial Cells (HUVEC) were obtained from Department of Cell Biology, China Medical University, China, cultured in EGM‐2 Endothelial Cell Growth Medium‐2 (Gibco), supplemented with 10% FBS, 100 units/mL penicillin, and 100 *μ*g/mL streptomycin.

### Lentivirus production and GCC infection

The design of shRNA sequences directed against DLL4 and lentivirus construction were carried out by Genepharma (Shanghai, China). GCCs were infected, and stable transfectants were selected in puromycin for 7 days. Afterward, GCCs were expanded to assess DLL4 downregulation.

### Western blotting

GCCs were washed in ice‐cold phosphate‐buffered saline (PBS) twice and collected using a cell scraper. 50 *μ*L of RIPA buffer, supplemented with 1 mmol/L PMSF, 1 *μ*g/mL leupeptin, 1 mmol/L *β*‐glycerophosphate, 2.5 mmol/L sodium pyrophosphate, and 1 mmol/L Na3VO4, were added on ice for 20 min, followed by centrifugation for 20 min at 12,000*g* and 4°C. Next, 50 *μ*g total protein from each sample were resolved on 10% sodium dodecyl sulfate (SDS) polyacrylamide gels and transferred onto polyvinylidene fluoride (PVDF) membranes. After blocking in TBST containing 4% skim milk for 2 h at room temperature, membranes were incubated with rabbit polyclonal anti‐DLL4 antibodies (diluted 1:4000; Abcam) in TBST overnight at 4°C. The membranes were then washed three times in TBST, and incubated in horseradish peroxidase (HRP)‐conjugated secondary antibodies (diluted 1:5000; Santa Cruz, Dallas, TX) in TBST for 2 h at room temperature. Detection was carried out by chemiluminescence using the ECL solution (Pierce, Rockford, IL).

### FACS analysis

Surface staining of the GCSPC‐related protein Nestin was analyzed by Fluorescence‐assisted cell sorting (FACS). Briefly, GCCs were harvested and fixed for 30 min. Cells were then washed twice with ice‐cold PBS supplemented with 1% bovine serum albumin (BSA). Incubation was carried out with mouse anti‐Nestin monoclonal antibody (diluted 1:400; Chemicon) for 30 min at 4°C in the dark. GCCs were further incubated with FITC‐conjugated donkey anti‐mouse secondary antibody (diluted 1:500; Santa Cruz) for 30 min 4°C in the dark, and cells surface staining was determined by flow cytometry (FACS Caliber, BD).

### Clonogenic assays

Cells were plated at 100 cells/well in 6‐well culture plates for 14 days in DMEM/F12 supplemented with 10% FBS, and colonies were fixed with 4% paraformaldehyde and stained with 1% crystal violet. Images were acquired on a digital microscope (80i, Nikon). The colonies formed (>2 mm diameter) were counted manually by three independent researchers.

### Tumorsphere culture

GCSPC sphere medium was described previously 14, and consisted of serum‐free DMEM/F12 (Invitrogen, Carlsbad, CA), 1 × B27 supplement (Invitrogen), 1 × N2 supplement (Invitrogen), 50 ng/mL epidermal growth factor (EGF; Peprotech), 100 ng/mL basic fibroblast growth factor (bFGF; Peprotech), 10 nmol/L gastrin (Sigma‐Aldrich), and 100 ng/mL noggin (Peprotech). For tumorsphere culture, cells were diluted in GCSPC sphere medium (5000 cells/mL), plated at 500 *μ*L per well in ultralow attachment 24 well plates (Corning), and provided 50 *μ*L GCSPC sphere medium every other day for 7 days. Afterward, spheres >100 *μ*m were counted as tumor sphere‐forming units. Tumorspheres were collected after filtering through a 70‐*μ*m strainer (BD) and submitted to immunofluorescence staining.

### Tumorsphere immunofluorescence staining

Spheroids were fixed with 4% paraformaldehyde for 24 h, dehydrated through a graded sucrose series, paraffin embedded, and sectioned. Four‐micron‐thick sections were blocked with 10% goat serum for 30 min at room temperature, and incubated with rabbit polyclonal anti‐DLL4 (diluted 1:1000; Abcam) and mouse monoclonal anti‐mucin 5AC (diluted 1:100; Santa Cruz) antibodies, respectively, for 1 h. After three washes with PBS, the sections were incubated with the corresponding secondary Alexa Fluor 488 or 594 secondary antibodies (all diluted 1:200, Invitrogen). Nuclei were counterstained with 4′,6‐diamidino‐2‐phenylindole (DAPI; Sigma‐Aldrich). Fluorescence was visualized on an immunofluorescence microscope (FV‐1000, Olympus, Japan). In all experiments, at least 10 spheroids per group were analyzed.

### Immunofluorescence

Kato III cells were fixed for 30 min in prechilled paraformaldehyde and permeabilized with 0.15% Triton X‐100. The cells were incubated overnight at 4°C with mouse monoclonal anti‐DLL4 (diluted 1:400; Abcam), mouse monoclonal anti‐Mucin 5AC (diluted 1:400; Santa Cruz), or rabbit polyclonal NICD‐1 (diluted 1:200; Abcam) antibodies, and subsequently incubated in the presence of secondary Alexa Fluor 594‐ or Alexa Fluor 488‐conjugated secondary antibodies (all diluted 1:200; Invitrogen). Nuclei were counterstained with DAPI (Sigma‐Aldrich). Fluorescence was visualized on an immunofluorescence microscope (FV‐1000; Olympus).

### Real‐time PCR

Total RNA was extracted from cultured cells using TRIzol Reagent (Invitrogen) and reverse‐transcribed with the Transcriptor First Strand cDNA synthesis kit (Invitrogen), following the manufacturer's instructions. Quantitative real‐time PCR was performed on an iCycler iQ Real‐Time PCR Detection System (Bio‐Rad, Hercules, CA) using iQ SYBR Green Supermix (Bio‐Rad). Real‐time PCR data were expressed as relative mRNA expression quantified with the Bio‐Rad iCycler system software and normalized to *β*‐actin levels. The forward and reverse primers used are listed in Table 1.

**Table 1:** The forward and reverse primers used in real‐time PCR

Gene	Sequence
JAG1	Forward: GTCCATGCAGAACGTGAACG
	Reverse: GCGGGACTGATACTCCTTGA
JAG2	Forward: TGGGCGGCAACTCCTTCTA
	Reverse: GCCTCCACGATGAGGGTAAA
DLL1	Forward: GATTCTCCTGATGACCTCGCA
	Reverse: TCCGTAGTAGTGTTCGTCACA
DLL3	Forward: CACTCCCGGATGCACTCAAC
	Reverse: GATTCCAATCTACGGACGAGC
DLL4	Forward: GTCTCCACGCCGGTATTGG
	Reverse: CAGGTGAAATTGAAGGGCAGT
HES1	Forward: TCAACACGACACCGGATAAAC
	Reverse: GCCGCGAGCTATCTTTCTTCA
HEY1	Forward: GTTCGGCTCTAGGTTCCATGT
	Reverse: CGTCGGCGCTTCTCAATTATTC

### MTT assay

Kato III cell viability was assessed using an MTT [3‐(4, 5‐dimethylthiazol‐2‐yl)‐2, 5‐diphenyltrazoliumbromide] assay. Approximately 1 × 10^4^ cells/well were seeded into 96‐well culture plates and cultured in DMEM with 10% FBS for 0–48 h, in the presence of 50 *μ*g/mL 5‐FU. After incubation, 20 *μ*L MTT (10 mg/mL) were added for 4 h at 37°C, and the medium was careful removed. 200 *μ*L DMSO were used to solubilize the formazan product for 20 min at room temperature, and optical density (OD) was read on a spectrophotometer (Bio‐800, Bio‐Rad) at 570 nm. Untreated Kato III cells cultured with serum‐free DMEM medium were used as a control group. Cell proliferation was calculated using the following equation:

$$\begin{array}{cc} \text{Cell proliferation rate}(\%) & =\text{OD (Experimental group}\\ & -\text{Control group)}/\text{OD control group}\\ & \times 100\% \end{array}$$

### Annexin V/PI apoptosis analysis

Apoptosis of Kato III cell was detected using an annexinV‐propidium iodide (PI) apoptosis detection kit (Becton Dickinson, Franklin Lakes, NJ) according to the manufacturer's instructions. Briefly, cells were resuspended in 1 × binding buffer at a concentration of 1 × 10^6^/mL. Then 5 *μ*L annexin V‐FITC antibody and 5 *μ*L PI were added in the dark at room temperature for 15 min. After washing twice with 1× binding buffer, cell‐surface staining was analyzed by flow cytometry.

(FACS Caliber, Becton Dickinson). KATO III cells in the upper‐right quadrant and the lower‐right quadrant showed late‐stage and early‐stage apoptosis, respectively.

### Tube formation assay

Tube formation was evaluated by angiogenesis in vitro assay. Briefly, 150 *μ*L of growth factor reduced Matrigel Basement Membrane Matrix (BD Biosciences) was added onto precooled 48‐well tissue culture well plates and left to solidify at 37°C for 60 min. HUVECs (40,000 cells/well) were seeded onto the bottom well of a Boyden chamber containing the polymerized matrix and cocultured in the presence KATO III cells, plated on the upper chamber containing a 0.4 *μ*m polycarbonate membrane insert for about 18 h at 37°C. Tube formation was analyzed under an inverted microscope at 20× magnification by evaluating the formation of polyhedral closed structures delimiting a lumen and images were acquired by a digital camera (Nikon Coolpix995).

### Side population analysis and sorting

Sorting the side population of GCCs was described previously (14). Briefly, GCCs were harvested and resuspended at 1 × 10^6^ cells/mL in prewarmed 37°C DMEM/F12 with 1% FBS. The cells were then labeled with Hoechst 33342 (Sigma‐Aldrich) at a concentration of 5 *μ*g/mL. The labeled cells were incubated in the dark for 75 min in a 37°C water bath with intermittent mixing, either alone or with 75 *μ*mol/L verapamil (Sigma‐Aldrich). The cells were resuspended in ice‐cold PBS containing 1% FBS after staining and were maintained at 4°C until flow cytometry analysis. Stained cells were analyzed using a flow cytometer (FACS Aria II, BD).

### Cell invasion assay

Boyden chambers (BD) with 8‐*μ*m pore size polystyrene filter inserts for 24‐well plates were used according to the manufacturer's instructions. Briefly, the upper compartment was coated with 50 *μ*L Matrigel (diluted 1:2; BD), then 5 × 10^4^ GCCs in 300 *μ*L DMEM were seeded into the upper compartment of each chamber. The chambers were placed into wells containing 750 *μ*L of complete medium. The invasion chambers were incubated under indicated conditions for 24 h at 37°C. Following incubation, the inserts were fixed and stained, and the number of migrating cells was counted. Two independent experiments were each performed in duplicate. Images were collected and quantified using Image‐Pro Discovery software (80i; Nikon, Japan).

### In vivo ectopic xenograft model

Eight‐ to 10‐week‐old male BALB/c nude mice (weighing 18–20 g each) were obtained from Vital River (Beijing, China). All animal protocols received prior approval from the China Medical University Animal Ethics Committee, and all experiments were performed in accordance with relevant guidelines and regulations. For tumor xenograft, side population of KATO, KATO‐shDLL4, and KATO‐NC cells were injected to the lower dorsum of mice at a concentration of 1*10^4^ or 1*10^5^, respectively. Twenty mice were included in each of the three groups and then separated into two subgroups. Mice were sacrificed by cervical dislocation 30 days after inoculation. 10 min before sacrifice, mice were anesthetized via inhalation of isoflurance to reduce the suffering. Tumor formation status was then estimated by autopsy.

### Statistical analyses

All statistical analyses were performed using the SPSS 19.0 software (IBM, Chicago, IL). Overall survival rates were determined using Kaplan–Meier curves, with an event defined as a cancer‐related death. The log‐rank test was used to assess differences between the survival curves in different patient groups. In univariate analysis, continuous data obtained from two groups were analyzed by two‐tailed Student's t‐test, and those from three or more groups by analysis of variance (ANOVA). Categorical data were evaluated by two‐tailed *χ* ^2^ test. All data were expressed as mean ± standard deviation (SD). *P *<* *0.05 was considered statistically significant.

## Results

### DLL4 overexpression correlates with cancer stem cell related protein expression and results in poor prognosis in gastric cancer patients

**Figure 1:**
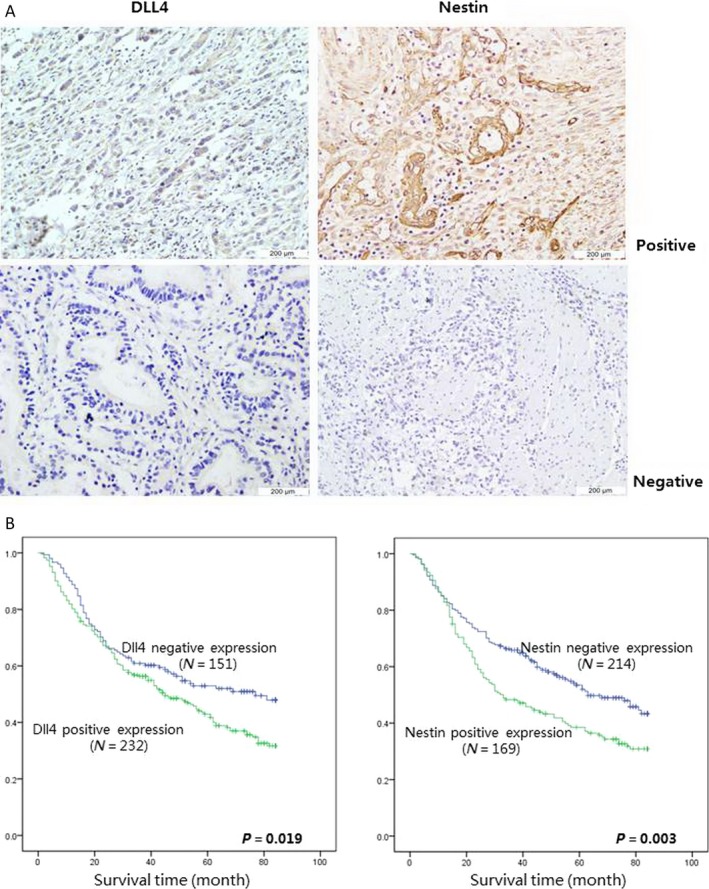
DLL4 overexpression correlates with cancer stem cell related protein expression and results in poor prognosis in gastric cancer patients. (A) DLL4 and Nestin expression were analyzed by immunohistochemical in 383 patients with gastric cancer. (The expressions were analyzed both in differentiated and undifferentiated gastric cancer, magnification 600 × ).(B) Overall survival rates from 383 gastric cancer patients were determined according to DLL4 (Left) and Nestin (Right) expression by Kaplan–Meier curves, respectively, with the event being defined as death related to cancer (151 patients showed negative expression and 232 patients showed positive expression of DLL4, 214 patients showed negative expression and 169 patients showed positive expression of Nestin). The log‐rank tests were used to identify differences between the survival curves of different patient groups (for dll4,left, *P* = 0.019, for Nestin right, *P* = 0.003).

DLL4 expression was assessed in 383 human gastric cancer tissue samples by immunohistochemical staining. Figure 1A illustrates DLL4 and Nestin expression in differentiated and undifferentiated gastric cancer cells. The correlation between DLL4 expression and the clinico‐pathological features in GC patients was analyzed using the *χ* ^2^ test. Interestingly, positive DLL4 expression was significantly associated with increased lymph node metastasis and distal metastasis risk as compared with patients showing negative DLL4 expression (Table 2). The association between DLL4 expression level and the cancer stem cell related protein Nestin was also analyzed. Positive expression of DLL4 was significantly correlated with that of Nestin (*P* < 0.05, Table 2).

**Table 2:** Association between DLL4 expression and clinic‐pathologic characteristics of patients with gastric cancer (*n* = 383)

	DLL4 expression		
	Negative(*n* = 151)	Positive(*n* = 232)	*P* value
Gender			0.97
Male	109	167	
Female	42	65	
Age			0.13
≤65	90	156	
>65	61	76	
Tumor size (cm)			0.13
≤5	76	135	
>5	75	97	
T category			0.34
T1	18	23	
T2	28	44	
T3	98	143	
T4	7	22	
N category			0.01^a^
N0	71	79	
N1	33	37	
N2	24	49	
N3	23	67	
Lauren grade			0.29
Intestinal	82	107	
Mix	21	38	
Diffuse	48	87	
Distant metastasis			0.01^a^
Negative	114	146	
Positive	37	86	
Nestin expression			0.01^a^
Negative	120	94	
Positive	31	138	

a *P* < 0.05.

Using Cox's proportional hazards regression model, the univariate relationships between tumor characteristics and patients' outcome were obtained (Table 3). Of the 383 patients analyzed, statistically significant differences in OS were seen, with a poor outcome for patients with higher staining of DLL4 and nestin. Other predictive factors that were found to be correlated with OS were T stage, N stage, and distant metastasis. Survival time analysis of the 383 involved patients by Kaplan–Meier curves revealed that DLL4‐negative patients displayed longer survival times compared to DLL4‐positive individuals (Fig. 1B, *P* = 0.019). Similarly, negative Nestin expression also correlated with significant survival benefits, as analyzed by log‐rank test (Fig. 1B, *P* = 0.003).

**Table 3:** Multivariate analysis of overall survival after surgery

	HR(95% CI)	*P* value
Age	1.331 (0.944–1.899)	0.116
Gender	1.177 (0.812–1.601)	0.342
Tumor size	1.078 (0.747–1.556)	0.692
T stage	1.930 (1.344–2.788)	<0.01^a^
N stage	1.434 (1.033–1.990)	<0.01^a^
Lauren grade	1.387 (1.178–1.666)	0.08
Distant metastasis	2.258 (1.774–3.108)	<0.01^a^
DLL4 expression	1.411 (1.120–1.925)	<0.01^a^
Nestin expression	1.397 (1.186–1.768)	0.017^a^

a *P* < 0.05.

### DLL4 silencing reduces gastric cancer stem/progenitor cell ratio in Kato III cells

**Figure 2:**
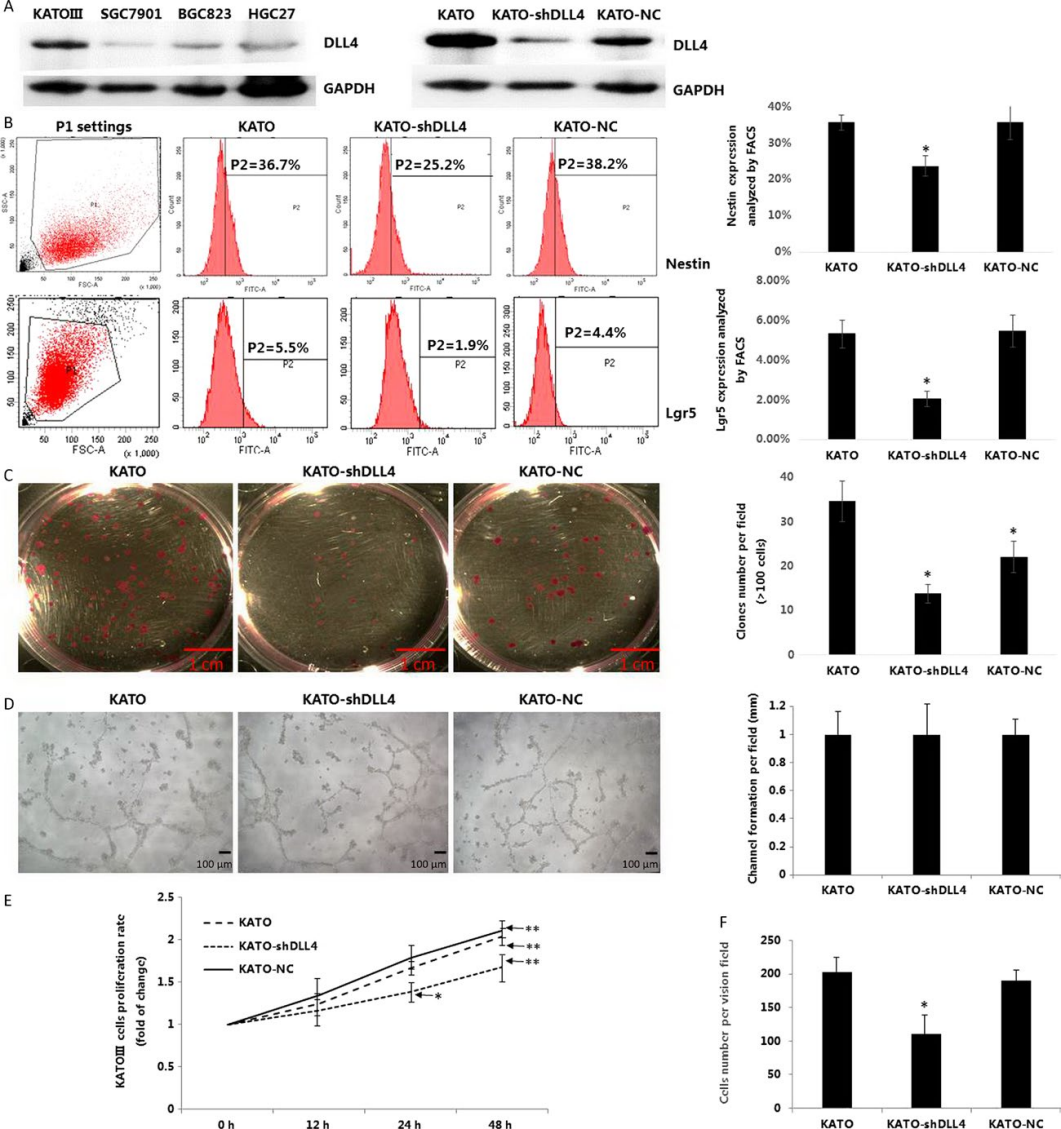
DLL4 silencing reduces gastric cancer stem/progenitor cell ratio in Kato III cells.(A) DLL4 expression was analyzed in four kinds of gastric cancer lines by Western blot. (Left). KATO were stably transfected with DLL4 shRNA (KATO‐shDLL4) or negative control shRNA (KATO‐NC), DLL4 expression were analyzed by Western blot (Right), both GAPDH served as a protein loading control. (B) Surface staining of cancer stem cells related proteins Nestin and lgr5 were analyzed by fluorescence‐assisted cell sorting (FACS, ratio of positive‐stained cell were expressed as mean ± SD, *n* = 4, both *P < 0.05). (C) Side population of KATO, KATO‐shDLL4 and KATO‐NC were plated at 100 cells/well in 6‐well culture plates for 14 days with DMEM/F12 supplemented with 10% FBS, colonies were fixed and stained. The number of colonies formed (magnification 40×, >2 mm diameter) was counted manually by three independent researchers (number of colons were expressed as mean ± SD, *n* = 6, *P < 0.05). **(**D) 150 *μ*L of growth factor reduced Matrigel Basement Membrane Matrix was added onto precooled 48‐well tissue culture well plates and left to solidify at 37°C for 60 min. HUVECs (40,000 cells/well) were seeded onto the bottom well of a Boyden chamber containing the polymerized matrix and cocultured in the presence of KATO, KATO‐shDLL4, and KATO‐NC cells, plated on the upper chamber containing a 0.4 *μ*m polycarbonate membrane insert for about 18 h at 37°C. Tube formation was analyzed under an inverted microscope. (magnification 200×, Length of tube were expressed as mean ± SD, *n* = 3). (E) KATO, KATO‐shDLL4, and KATO‐NC cells were plated at 1 × 104 cells/well in 96‐well culture plates and cultured in DMEM with 10% FBS for 0–48 h. After incubation, 20 *μ*L MTT (10 mg/mL) were added for 4 h at 37°C, and the medium was careful removed. 200 *μ*L DMSO were used to solubilize the formazan product for 20 min at room temperature, and OD was read on a spectrophotometer (Bio‐800; Bio‐Rad) at 570 nm. Cell proliferation was calculated using the following equation: Cell proliferation rate (%) = OD (Experimental group – Control group)/OD control group × 100%. (F) KATO, KATO‐shDLL4, and KATO‐NC cells were fixed and cultured, invasion ability were analyzed by Boyden chamber invasion assay, cells were fixed with 4% paraformaldehyde and stained with 1% crystal violet. (Cell number per field were expressed as mean ± SD, *N* = 4, *P < 0.05). DMEM, Dulbecco's Modified Eagle's medium; FBS, fetal bovine serum; OD, optical density.

To investigate the correlation between DLL4 expression and GCSPCs, we first measured DLL4 expression in four gastric cancer cell lines, including KATOIII, SGC7901, BGC823, and HGC27. The most obvious DLL4 expression was observed in KATOIII. Next, KATOIII cells were stably transfected with shDLL4 (KATO‐shDLL4) and negative control shRNAs (KATO‐NC). Western blotting confirmed that DLL4 expression was effectively inhibited in KATO‐shDLL4 cells (Fig. 2A). We then assessed the surface expression of Nestin and Lgr5, two confirmed specific markers of GCSPCs. As shown in Figure 2B, KATO‐shDLL4 cells had lower Nestin and Lgr5 expression levels compared with nontranfected KATO cells (1.5‐ and 2.6‐fold decrease, respectively; both *P* < 0.05). In contrast, levels of Nestin and Lgr5 in KATO‐NC and KATO cells were similar (*P* > 0.05). We further evaluated the variation in stem/progenitor cells by clonogenic assays. As shown in Figure 2C, KATO‐shDLL4 cells formed fewer and smaller clones compared with KATO cells (13.9 ± 2.1 vs. 34.7 ± 4.5, *P* < 0.05). To better understand the overall tumor phenotype among KATO III cell groups, angiogenesis, proliferation, and invasion ability were also analyzed. As shown in Figure 2D, There were no significant difference in angiogenesis ability among three groups (all *P* > 0.05). However, we find significant decrease in both proliferation and invasion ability compared with KATO cells (both *P* < 0.05).

### DLL4 knockdown inhibits gastric cancer stem/progenitor cell self‐renewal but enhances multidifferentiation ability

**Figure 3:**
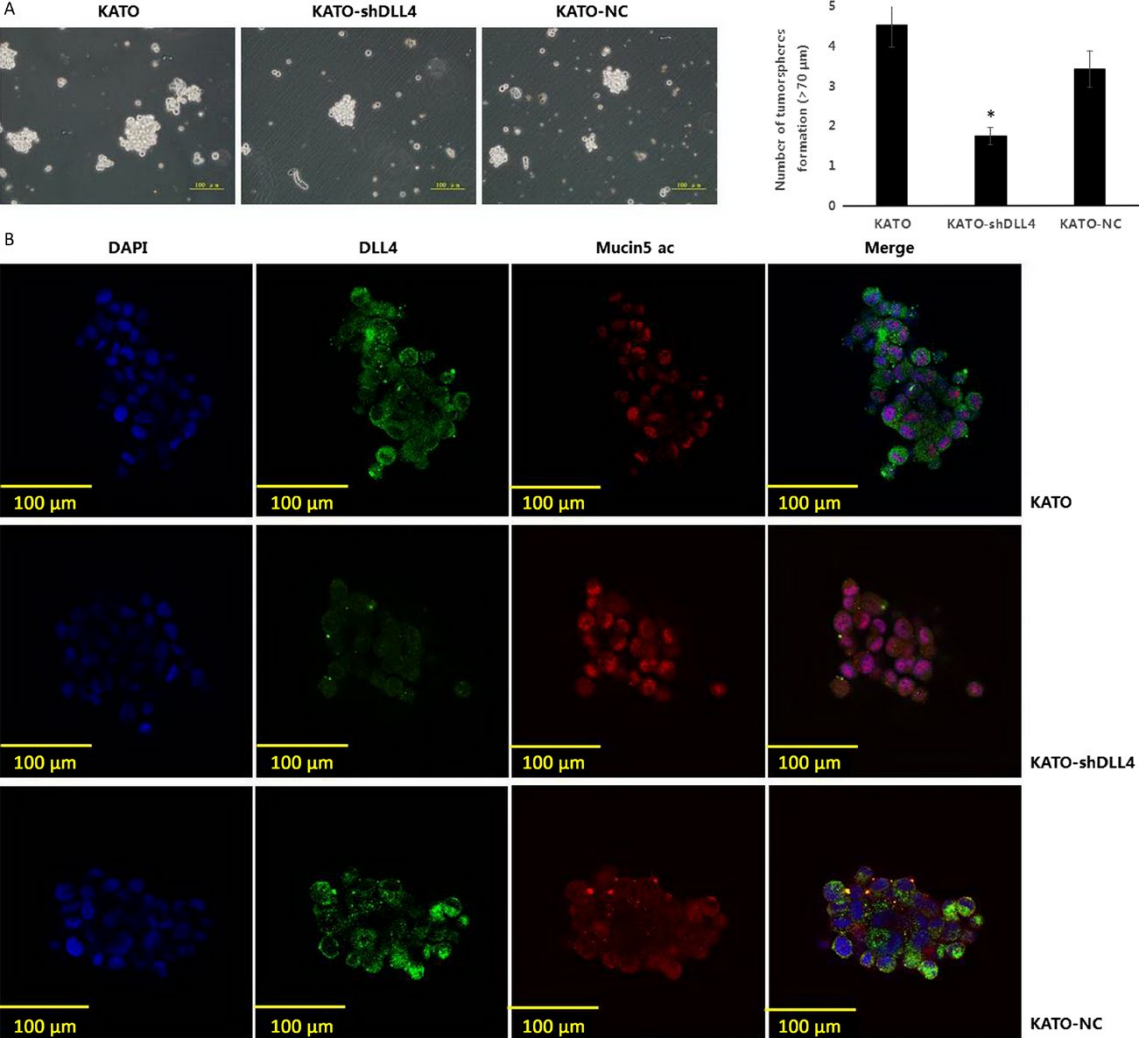
DLL4 knockdown inhibits gastric cancer stem/progenitor cell self‐renewal but enhances multidifferentiation ability. (A) KATO, KATO‐shDLL4, and KATO‐NC were diluted in GCSPC sphere medium (5000 cells/mL) and plated at 500 *μ*L per well in ultralow attachment 24 well plates. (magnification 60×, numbers of tumorsphere were expressed as mean ± SD, *n* = 3, *P < 0.05). **(**B) Spheroids of KATO, KATO‐shDLL4, and KATO‐NC were fixed and sectioned, DLL4 and stem/progenitor cell differentiation protein Mucin5 ac expression were analyzed by immunofluorescence staining (DAPI showed blue staining, DLL4 showed green staining, and Mucin5 ac showed red staining, magnification 600×). GCSPC, Gastric cancer stem/progenitor cells.

To assess the self‐renewal and differentiation capacities of GCSPCs, the three KATO cell lines were tested on their ability to form tumorshperes. As shown in Figure 3A, significantly less tumor spheroids were found in KATO‐shDLL4 cultures compared with KATO cultures (1.8 ± 0.2 vs. 4.5 ± 0.5, *P* < 0.05). Next, we compared the levels of DLL4 and Mucin 5AC, a specific marker of GCSPC differentiation, by confocal imaging. As shown in Figure 3B, spheroids in KATO‐shDLL4 culture exhibited reduced DLL4 expression compared with those of the KATO group. In contrast, markedly increased Mucin 5AC expression was observed in spheroids in KATO‐shDLL4 culture compared with those in the KATO group.

### DLL4 knockdown inhibits Notch‐1 signaling pathway activation in GCSPCs

**Figure 4:**
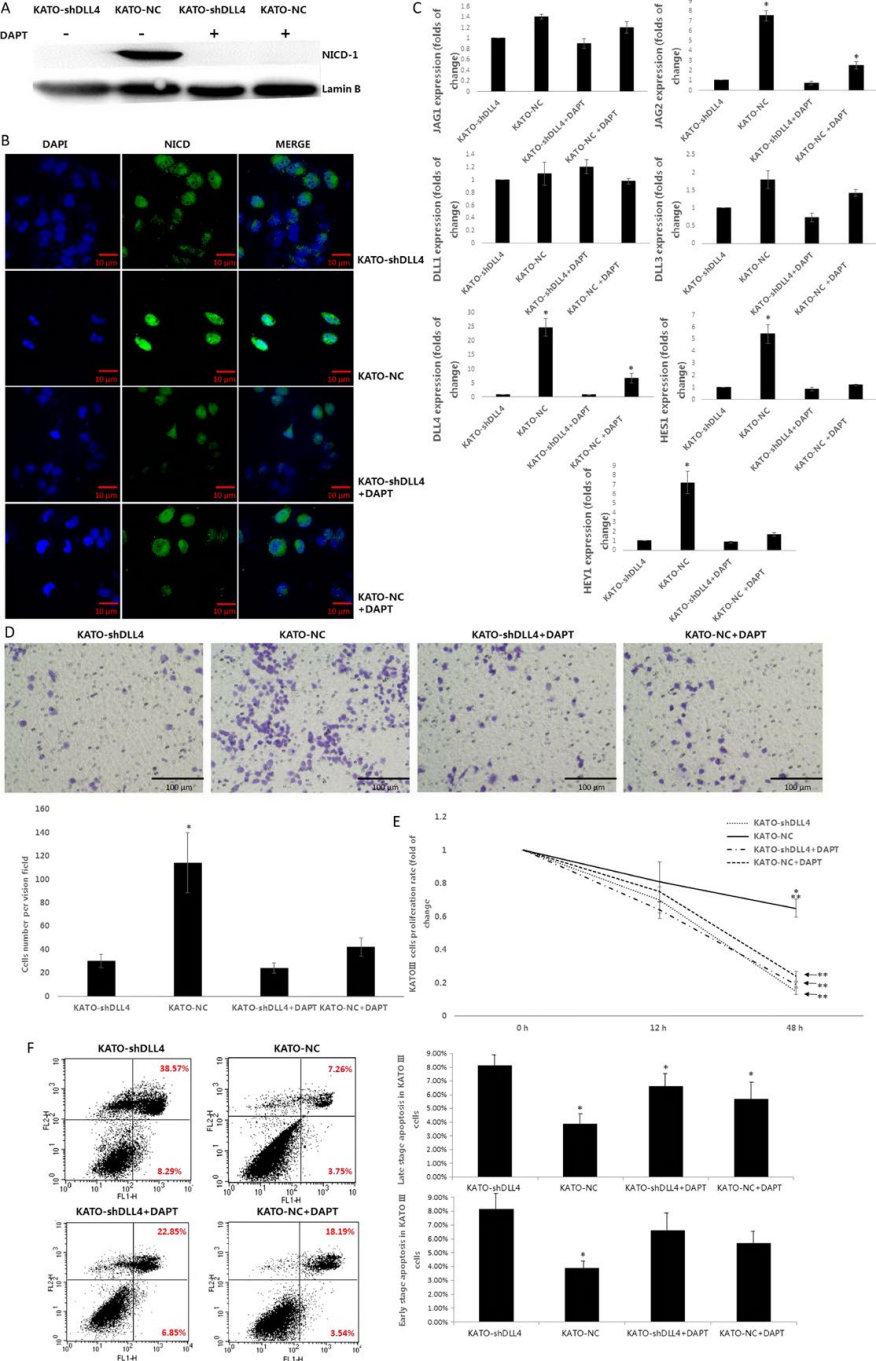
DLL4 knockdown inhibits Notch‐1 signaling pathway activation in gastric cancer stem/progenitor cells. (A) Specific Notch‐1 pathway protein NICD‐1 expression was analyzed in KATO‐shDLL4 and KATO‐NC by Western blot, with or without Notch‐1 pathway inhibitor DAPT added, Lamin B served as a protein loading control. **(**B) NICD expression was analyzed by immunofluorescence staining (DAPI showed blue staining, NICD showed green staining, magnification 400×).(C) Relative JAG1, JAG2, DLL1, DLL3, DLL4 mRNA expression quantified with the Bio‐Rad iCycler system software and normalized to *β*‐actin levels. (*P < 0.05). (D) KATO‐shDLL4, KATO‐NC, KATO‐shDLL4 + DAPT, and KATO‐NC+DAPT were fixed and cultured, invasion ability were analyzed by Boyden chamber invasion assay, cells were fixed with 4% paraformaldehyde and stained with 1% crystal violet. (magnification 200×, Cell number per field were expressed as mean ± SD, *N* = 4, *P < 0.05). **(**E) KATO‐shDLL4, KATO‐NC, KATO‐shDLL4 + DAPT, and KATO‐NC+DAPT were plated at 1 × 10^4^ cells/well in 96‐well culture plates and cultured in DMEM with 10% FBS for 12–72 h, in the presence of 50 *μ*g/mL 5‐FU. After incubation, 20 *μ*L MTT (10 mg/ml) were added for 4 h at 37°C, and the medium was careful removed. 200 *μ*L DMSO were used to solubilize the formazan product for 20 min at room temperature, and OD was read on a spectrophotometer (Bio‐800, Bio‐Rad) at 570 nm. Cell proliferation was calculated using the following equation: Cell proliferation rate (%) = OD (Experimental group–Control group)/OD control group × 100%. F. KATO‐shDLL4, KATO‐NC, KATO‐shDLL4 + DAPT, and KATO‐NC+DAPT cells were resuspended in 1× binding buffer at a concentration of 1 × 10^6^/mL. Then 5 *μ*L annexin V‐FITC antibody and 5 *μ*L PI were added in the dark at room temperature for 15 min. After washing twice with 1× binding buffer, cell‐surface staining was analyzed by flow cytometry. Cells in the upper‐right quadrant and the lower‐right quadrant showed late‐stage and early‐stage apoptosis, respectively. (Numbers of cells were expressed as mean ± SD, *n* = 3, *P < 0.05). DMEM, Dulbecco's Modified Eagle's medium; FBS, fetal bovine serum; OD, optical density.

Western blotting was used to assess the association of DLL4 expression and activation of the Notch‐1 pathway. The expression of NICD‐1, a specific Notch‐1 pathway effector, was compared between KATO‐NC and KATO‐shDLL4 cells with or without DAPT, a common Notch‐1 pathway inhibitor. As shown in Figure 4A, KATO‐NC cells exhibited elevated NICD‐1 expression compared with KATO‐shDLL4 cells. When DAPT was added to both cell cultures, NICD‐1 expression was remarkably suppressed in KATO‐NC cells. To confirm Western blotting data, NICD‐1 protein levels were also determined by immunofluorescence. As shown in Figure 4B, enhanced NICD‐1 expression levels were observed in KATO‐NC cells. Of note, the NICD‐1 protein was predominantly localized to the nucleus in all conditions, indicating that DLL4 shRNA and DAPT have similar effects on the Notch‐1 pathway. We next used real‐time PCR to quantitatively analyze mRNA levels of five iconic markers (JAG1, JAG2, DLL1, DLL3, DLL4) and two specific target genes (HES1 and HEY1) of the Notch‐1 pathway. As shown in Figure 4C, JAG2, DLL4, HES1, and HEY1 levels were all increased in KATO‐NC cells compared with the values obtained for KATO‐shDLL4 cells(7.5‐, 24.7‐, 5.4‐, and 7.2‐fold increase, respectively; all *P* < 0.05).

As a measure of cell invasiveness, the ability of KATO cells to cross a matrigel‐coated insert was assessed under different conditions. KATO‐NC cells showed greater invasion ability compared with KATO‐shDLL4 cells (3.8‐fold increase, *P* < 0.05). Notably, the invasion abilities of KATO‐NC+DAPT and KATO‐shDLL4 + DAPT were not significantly different from values obtained for KATO‐shDLL4 (Fig. 4D). The resistance of the different KATO cells to 5‐FU chemotherapy was evaluated by MTT assay. As shown in Figure 4E, KATO‐NC cells exhibited a lower rate of cell death than KATO‐shDLL4 48 h post 5‐FU treatment (4.3‐fold increase, *P* < 0.05). Meanwhile, cytotoxicity rates of KATO‐NC+DAPT and KATO‐shDLL4 + DAPT were similar to the values obtained for untreated KATO‐shDLL4. Then cells apoptosis were analyzed by AnnexinV/PI cell‐surface staining, KATO‐NC, KATO‐NC+DAPT, and KATO‐shDLL4 + DAPT all exhibited a decreased late stage apoptosis compared with KATO‐shDLL4 cells (51.24%, 20.18%, and 27.45% decreased, respectively, all *P* < 0.05). However, only KATO‐NC reach a significant decrease compared with KATO‐shDLL4 cells in early‐stage apoptosis (47.22% decrease, *P* < 0.05).

### DLL4 silencing in GCSPCs reduces tumor formation ability in vivo

The effect of DLL4 silencing on tumor formation ability in vivo was evaluated. Side population of KATO, KATO‐shDLL4, and KATO‐NC cells were isolated by FACS. Nude mice were injected with KATO_sp_, KATO‐shDLL4_sp_, and KATO‐NC_sp_ cells, respectively. To obtain rigorous results, each group was separated into two subgroups and treated with 1*10^4^ or 1*10^5^ cells. As shown in Table 4, tumorigenicity was significantly reduced in mice injected with 1*10^4^ KATO‐shDLL4_sp_ cells compared with mice injected with 1*10^4^ KATO_sp_ or KATO‐NC_sp_ cells (3‐ and 2.5‐fold, respectively; both *P* < 0.05). At 1*10^5^ cell density, mice in the KATO‐shDLL4_sp_ subgroup still maintained lower tumor formation rate compared with the 100% tumor formation rate obtained for the KATO_sp_ and KATO‐NC_sp_ groups (1.4‐fold decrease, respectively; both *P* < 0.05).

**Table 4:** Nodules formation of mice in each groups

		KATO_sp_		KATO‐shDLL4_sp_		KATO‐NC_sp_		*P*‐value
		1 × 10^4^	1 × 10^5^	1 × 10^4^	1 × 10^5^	1 × 10^4^	1 × 10^5^	
Nodule formation	Yes	6	10	2	7	5	10	<0.05^a^
	No	4	0	8	3	5	0	<0.05^b^

a *P* < 0.05 compared at a density of 1 × 10^4^ among three groups.

b *P* < 0.05 compared at a density of 1 × 10^5^ among three groups.

## Discussion

Cancer stem/progenitor cells are a small cell population involved in cancer, with unique characteristics such as self‐renewal, proliferation, differentiation, and tumorigenicity 15. Multiple studies have reported that CSPCs are closely related to tumor growth, metastasis, and recurrence 16, inducing poor prognosis in different kinds of cancer 17, 18. So far, reports assessing CSPCs in gastric cancer are scarce, and the specific mechanism by which CSPCs contribute to gastric cancer remains to be characterized.

Delta‐Like ligand 4 (DLL4) is a major ligand for Notch receptors found on chromosome 15q21.1 19. Existing studies suggested that DLL4 facilitates tumor growth by accelerating vasculature generation 20, 21, 22. Recent reports have confirmed that DLL4 expression is tightly related with TNM stage, node stage, and distant metastasis, predicting poor prognosis in patients with nasopharyngeal carcinoma 23. In this study, we demonstrated that DLL4 overexpression associates with poor prognosis in gastric cancer by assessing tissue samples and patient survival (Fig. 1A). DLL4 overexpression largely correlated with shorter survival time (Fig. 1B). Simultaneously, clinico‐pathological features such as high TNM stage, node metastasis, and distant metastasis were more represented in patients with DLL4 overexpression (Table 2). These findings confirmed that DLL4 is a significant predictive factor of advanced‐stage gastric cancer and tumor metastasis and is closely related to clinical outcome. We also found that the stem/progenitor cell marker Nestin was expressed in cancerous tissue samples associated with a poor outcome (Fig. 1B). Interestingly, DLL4 and Nestin levels were tightly associated, suggesting DLL4 may affect GCSPCs through certain mechanisms in gastric cancer progression. Next, we evaluated specific effects of DLL4 in GCSPCs. As shown in Figure 2B, DLL4 silencing resulted in decreased levels of two stem/progenitor cell marhers: Nestin and Lgr5. The clone formation ability of CSPCs was markedly reduced by DLL4 knockdown (Fig. 2C), indicating that DLL4 repression reduced GCSPC ratio to a certain extent. CSPCs can advance to a heterogeneous lineage of cancer cells, ultimately forming tumors 24; therefore, we suggest that elevated DLL4 levels promote gastric CSPCs and tumor formation.

The tumorsphere culture assay revealed that CSPCs showed reduced self‐renewal ability and increased Mucin 5AC expression after DLL4 silencing (Fig. 3A and B). Mucin 5AC is a confirmed specific marker of multidifferentiation 25, so DLL4 silencing inhibited the self‐renewal ability of CSPCs and accelerated the differentiation process. Self‐renewal and differentiation are crucial characteristics of CSPCs 26, as cancer cell populations are stabilized by these features. Previous studies have identified a role for DLL4 in tumor angiogenesis 27. Our findings demonstrate that DLL4 overexpression enhances CSPC self‐renewal, stimulating tumor proliferation, while restraining CSPC differentiation, which may lead to undifferentiated gastric cancer and a poor prognosis.

Notch‐1 is one of the Notch pathway receptors. When Notch‐1 binds its ligands (JAG1, JAG2, DLL1, DLL3, and DLL4) two proteolytic cleavages (TACE and *γ*‐secretase) occur that release NICD‐1 to the nucleus. NICD‐1 combines with the RBPJ protein and induces the transcription of Notch target genes Hes‐1 and Hey‐1 28. The Notch signaling pathway is involved in many important processes including cell fate, proliferation, chemoresistance, and apoptosis 29. Here, the Notch inhibitor DAPT was assessed as a control, and we found that reduced DLL4 levels affected NICD‐1 activity comparatively to DAPT treatment, nearly blocking NICD‐1 release into the nucleus (Fig. 4A and B). The Notch target genes were repressed similarly (Fig. 4C), and the proliferation and drug‐resistance of CSPCs with DLL4 knockdown also showed a sharp decline (Fig. 4D and E). The effect of DLL4 silencing was similar to that of adding DAPT on Notch‐1 pathway activity, indicating that DLL4 impacts the biological characteristics of CSPCs through the activation of the Notch‐1 pathway activation.

Nestin, a cytoskeleton‐associated class VI IF protein, is a neuronal stem/progenitor cell marker that is expressed in progenitor cells of various tissues, including central nervous tumors 30, lung cancer, 31 and breast cancer 32. Recent reports support a link between nestin and malignant characteristics and suggest that abundant nestin expression is correlated with greater malignancy and poorer prognosis in different cancers. Our group also have shown that Nestin is an important stem cell related marker which was expressed in gastric cancer and correlated with a pessimistic prognosis. Here, we found that the stem/progenitor cell marker Nestin was expressed in cancerous tissue samples associated with a poor outcome (Fig. 1B). Interestingly, DLL4 and Nestin levels were tightly associated, suggesting DLL4 may affect GCSPCs through certain mechanisms in gastric cancer progression. Next, we evaluated specific effects of DLL4 in GCSPCs.

In summary, DLL4 is associated with CSPCs in gastric cancer, and its expression impacts CSPC stemness characteristics associated with the Notch‐1 pathway including self‐renewal, differentiation, proliferation, chemoresistance, and tumor formation. Our study provides a crucial supplement to the current knowledge of DLL4/Notch pathway in gastric cancer, and constitutes a potential reference for exploration and clinical therapy.

## Conflict of Interest

No potential conflicts of interest were disclosed.
